# Specific Roles of Akt iso Forms in Apoptosis and Axon Growth Regulation in Neurons

**DOI:** 10.1371/journal.pone.0032715

**Published:** 2012-04-11

**Authors:** Hector Diez, Juan Jose Garrido, Francisco Wandosell

**Affiliations:** 1 Centro de Investigación Biomédica en Red sobre Enfermedades Neurodegenerativas (CIBERNED) and Centro de Biología Molecular “Severo Ochoa", CSIC-UAM, Univ. Autonoma de Madrid, Madrid, Spain; 2 Laboratory of Neuronal Polarity, Department of Molecular, Cellular and Developmental Neurobiology, Instituto Cajal, CSIC, Madrid, Spain; Consejo Superior de Investigaciones Cientificas, Spain

## Abstract

Akt is a member of the AGC kinase family and consists of three isoforms. As one of the major regulators of the class I PI3 kinase pathway, it has a key role in the control of cell metabolism, growth, and survival. Although it has been extensively studied in the nervous system, we have only a faint knowledge of the specific role of each isoform in differentiated neurons. Here, we have used both cortical and hippocampal neuronal cultures to analyse their function. We characterized the expression and function of Akt isoforms, and some of their substrates along different stages of neuronal development using a specific shRNA approach to elucidate the involvement of each isoform in neuron viability, axon development, and cell signalling. Our results suggest that three Akt isoforms show substantial compensation in many processes. However, the disruption of Akt2 and Akt3 significantly reduced neuron viability and axon length. These changes correlated with a tendency to increase in active caspase 3 and a decrease in the phosphorylation of some elements of the mTORC1 pathway. Indeed, the decrease of Akt2 and more evident the inhibition of Akt3 reduced the expression and phosphorylation of S6. All these data indicate that Akt2 and Akt3 specifically regulate some aspects of apoptosis and cell growth in cultured neurons and may contribute to the understanding of mechanisms of neuron death and pathologies that show deregulated growth.

## Introduction

Neurons are perhaps one of the most challenging systems for molecular and cellular biology. Their physiological function is only possible because of their organization into morphological and functional differentiated compartments resulting from a precise coordination of extracellular and intracellular signalling pathways.

Akt (also known as Protein Kinase B– PKB) is a widely studied protein that belongs to the AGC family of serine/threonine kinases [1], showing a virtually ubiquitous distribution and playing a basic role in nervous system. Initially described as the human homolog of a viral oncogene [2], it is involved in many biological processes and pathologies, such as metabolism regulation, cell growth, survival, proliferation, cancer, and neurodegenerative disorders [3]. There are three Akt isoforms encoded by three different genes (Akt1/PKBα, Akt2/PKBβ, and Akt3/PKBγ). These paralogs are closely related and share a high homology at protein level [4]. Extracellular signals induce Akt activation through class I phosphoinositide 3-kinase (PI3K), for which it has been traditionally considered as the main effector [3]. In this pathway, the production of phosphatidylinositol (3,4,5)-triphosphate (PIP_3_) in the plasma membrane leads to Akt activation by phosphorylation through phosphoinositide-dependent kinase-1 (PDK1) and mammalian target of rapamycin complex 2 (mTORC2) in two amino acids residues, threonine 308 and serine 473, respectively (amino acid numbers corresponding to Akt1 isoform). [5]. Active Akt exerts its function through the phosphorylation of a wide range of substrates, including transcription factors as the FoxO family [6], kinases such as Glycogen Synthase Kinase 3 (GSK3) [7], or regulators of mTORC1 such as TSC2 [8] and PRAS40 [9].

Akt isoforms are differentially expressed and have been related to distinct functions. Akt1 and Akt2 are widely expressed and especially high levels of Akt2 are present in the heart, skeletal muscle, adipose tissue, and testes. Akt3 expression is mainly restricted to brain and testes, although it is also present in adipose tissue, mammal glands, and lungs [4]. Furthermore, each isoform is amplified in different cancer types [10]. Suppression of Akt isoforms in knockout mice also reveals distinct physiological functions for each isoform. Akt1 genetic ablation induces a reduction of body and cell size [11], [12], Akt2 knockouts show diabetes mellitus-like syndrome [13], [14], and Akt3 deletion causes smaller brain size and *corpus callosum* disorganization [15], [16].

Akt is involved in many physiological functions in the nervous system, including the regulation of neuron survival [17], [18], [19], estradiol and IGF-1 induced neuroprotection [20], [21] and the inhibition of GSK3, which plays a major role in physiological and pathological conditions in brain.[17]. GSK3 presents two ubiquitous isoforms coded by two different genes (GSK3α and GSK3β). Moreover, GSK3β has two splicing variants, GSK3β1 and GSK3β2, which are highly expressed in the nervous system and are involved in the regulation of multiple functions [7], including cell survival, metabolism and cell growth. Akt inhibits GSK3α/β by phosphorylation of serine residues in the amino-terminal region (serine 21 in α and serine 9 in β), which are inserted into the kinase domain of GSK3, hindering the entry of substrates [22]. Akt also plays an important role in development, as it has been linked to neurogenesis [23] and to axon establishment and elongation through the regulation of GSK3 [24], [25], [26], [27], [28]. Altered Akt function has been associated to pathologies such as Huntington's disease [29], [30], Alzheimer's disease [31], spinocerebellar ataxia type 1 [32], schizophrenia [33], or autism [34]. In glial cells, Akt3 has an important role in oligodendrocyte genesis [15], [16] and a recent publication has related Akt isoforms to astrocyte growth and glioma genesis in PTEN and p53 null conditions [35].

Another substrate downstream the Akt pathway, mTORC1, is a master regulator of metabolism, translational control, and cell growth [36], and has also been involved in axon development and growth [37], [38], [39] and in neurogenesis [40]. It has been proposed that this process is regulated by the most studied substrates of mTORC1, S6K1 [38] and 4EBP1 [37]. S6K1, a member of the AGC kinase family, is involved in cell growth, insulin resistance and regulation of protein translation through the ribosomal S6 protein [41], while 4EBP1 impairs eIF4E function and plays a role in the repression of translation initiation [42]. Their role in axon development has been proposed to be related to their function in the regulation of the synthesis of proteins indispensable for axon development [37], [38].

We have only a faint knowledge of the specific function of each Akt isoform in differented neurons. Here, we shed light on their roles in neurons, using primary *in vitro* neuronal cultures widely used to study the cell biology of the neuron. Primary neuron culture allows for the analysis of isolated neurons under controlled conditions without the interference of systemic perturbations [43]. They are also an established model of neuron development, neuronal polarity, axonogenesis and dendritogenesis, or ionic channel physiology [44] and have been largely used in studies of neuron death [19].

We have characterized the evolution of Akt isoform expression and function during the development of cortical and hippocampal neurons in culture. For this purpose, we have used a short hairpain RNA interference (shRNA) approach to analyse their involvement in two of the main cellular processes regulated by Akt: cell viability and axon growth. Finally, we describe how cellular signalling is differentially regulated in neurons lacking one or another Akt isoform by the action of specific interference shRNAs.. Our results might help to understand the molecular basis of each Akt isoform function in neuronal development and survival.

## Results

### Characterization of Akt isoforms and Akt activity in neuronal cell culture development

**Figure 1:**
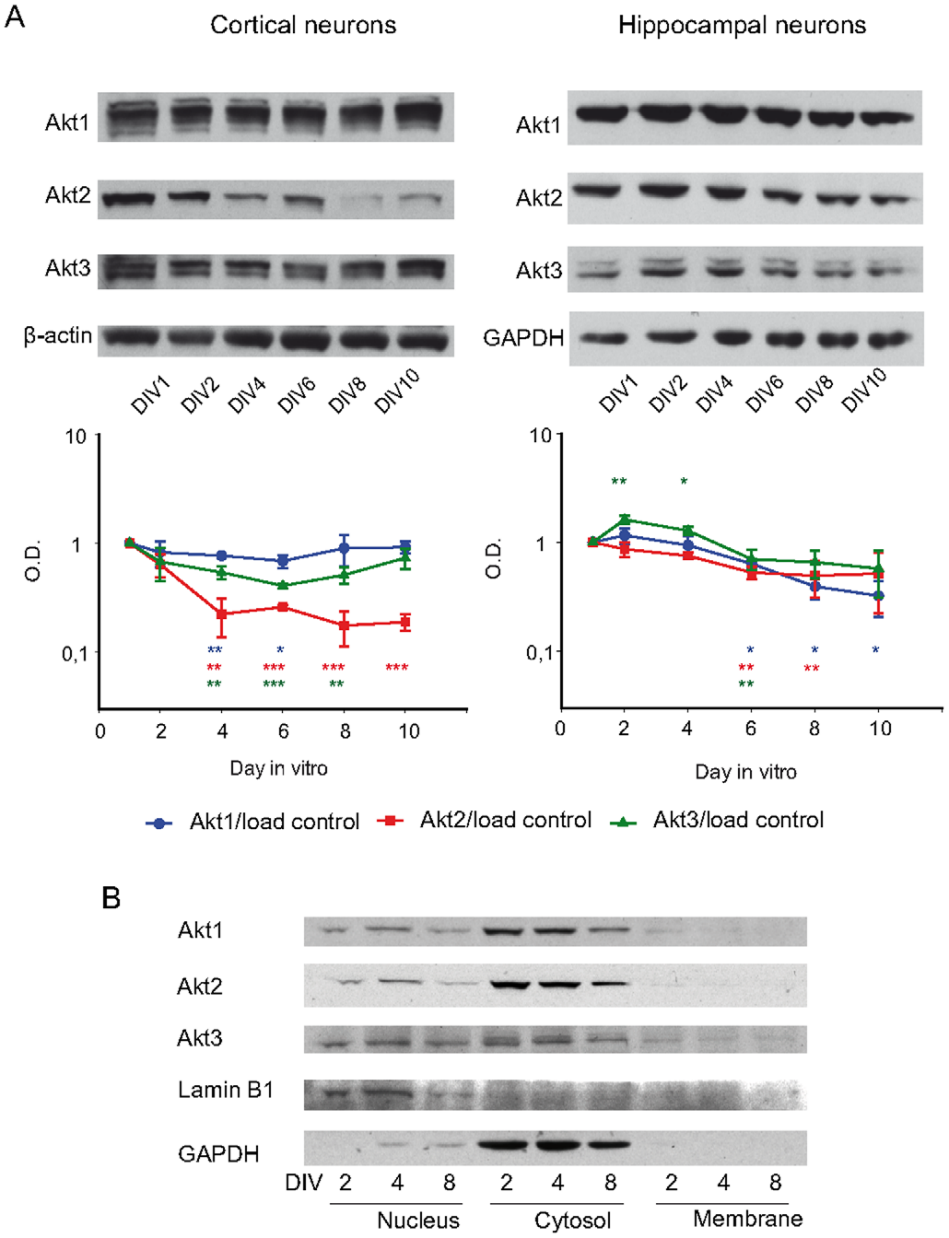
Akt isoforms levels change during neuron development. **A.** *Ex vivo* evolution of the isoforms of Akt in cortical (left) and hippocampal (right) neuron cultures at 1, 2, 4, 6, 8 and 10 days *in vitro* (DIV). Cell extracts were obtained as described in Methods, and Western blots analysed with specific antibodies against each isoform. β-actin was used as loading control for cortical neurons and GAPDH for hippocampal neurons. Western blots were quantified and normalised with respect to the control protein as indicated. The data from 1 DIV was always considered as 1 relative unit and the values are represented in logarithmic scale. The graphs represent three independent experiments (each point represents mean ± SEM). Samples were compared to 1 DIV using Student's t test; *: *p*<0.05; **: *p*<0.01; ***: *p*<0.001. In cortical neurons Akt2 and Akt3 levels were reduced from 4 DIV on and Akt1 changes slightly at 4 DIV–6 DIV. In hippocampal neurons, Akt1 levels decreased from 6 DIV on, Akt2 showed a tendency to reduction from 4 DIV on (which is statistically significant at 4 DIV and 6 DIV) an Akt3 is slightly increased at 2–4 DIV. **B.** Subcellular distribution of Akt isoforms. Total extract from cortical neurons was fractionated as indicated in Methods. For each sample (membrane, citosol and nucleus) the presence of Akt isoforms was determined by Western blot. Lamin B1 was used as marker and loading control for the nuclear fraction and GAPDH for the cytosolic fraction. Note that Akt3 showed a relatively higher nuclear proportion than the other isoforms.

In order to understand the role of each Akt isoform in neuronal development, first we analysed the expression pattern of each isoform during development in cultured embryonic cortical and hippocampal mouse neurons. Thus, we cultured both types of neurons for different days in vitro (DIV) and protein extracts were obtained from 1, 2, 4, 6, 8 and 10 DIV neurons. The expression of Akt 1, Akt2 and Akt3 along neuronal development at the days indicated above was studied using specific antibodies against each isoform (**Figure 1**). The data were normalised with respect to total protein, GAPDH or β-actin, and represented as relative units. In all cases the amount of 1 DIV was arbitrarily considered as 1 relative unit (**Figure 1A**).

In cortical neurons, Akt1 levels were relatively stable, with only faint but significant changes at 4 DIV and 6 DIV. Akt3 levels were also significantly reduced from 4 DIV to 8 DIV, but returned to 1 DIV levels at 10 DIV as observed for Akt1. Interestingly, Akt2 expression levels were very significantly reduced at 4 DIV and Akt2 expression was only 22.2±8.66% (p<0.01) of that observed at 1 DIV (**Figure 1A**). This reduction was maintained until 10 DIV (6 DIV: 25.8±1.31%, *p*<0.001; 8 DIV: 17.5±6.17%, *p*<0.001; 10 DIV: 18.8±3.29%, *p*<0.001).

In contrast, the levels of the three Akt isoforms in hippocampal neurons showed a tendency to reduction from 6 DIV, which was statically significant (*p*<0.01) for Akt2 at 4 DIV and 6 DIV and for Akt1 from 4 DIV on. Surprisingly, Akt3 levels were transiently increased at 2 DIV and 4 DIV when compared with 1 DIV (2 DIV: 161.6±14.4%, *p*<0.01; 4 DIV: 127.6±11.9%, *p*<0.05) (**Figure 1A**), even if Akt3 levels were also significantly reduced at 6 DIV. Next, we aimed at determining the cellular location of each Akt isoform in neurons. However, no appropriate antibodies for immunocytological analysis were found. Consequently, we tried to obtain some indirect information using subcellular fractionation methods with cortical primary cultures (**Figure 1B**). Antibodies against GAPDH and the nuclear protein Lamin B1 were used as internal controls for cytosolic and nuclear fractions, respectively. Western blots results showed that all Akt isoforms were mostly present in the cytosolic fraction. Interestingly, Akt3 showed a higher nuclear to cytosol ratio. In contrast, the three isoforms were barely detected in the membrane fraction (**Figure 1B**).

**Figure 2:**
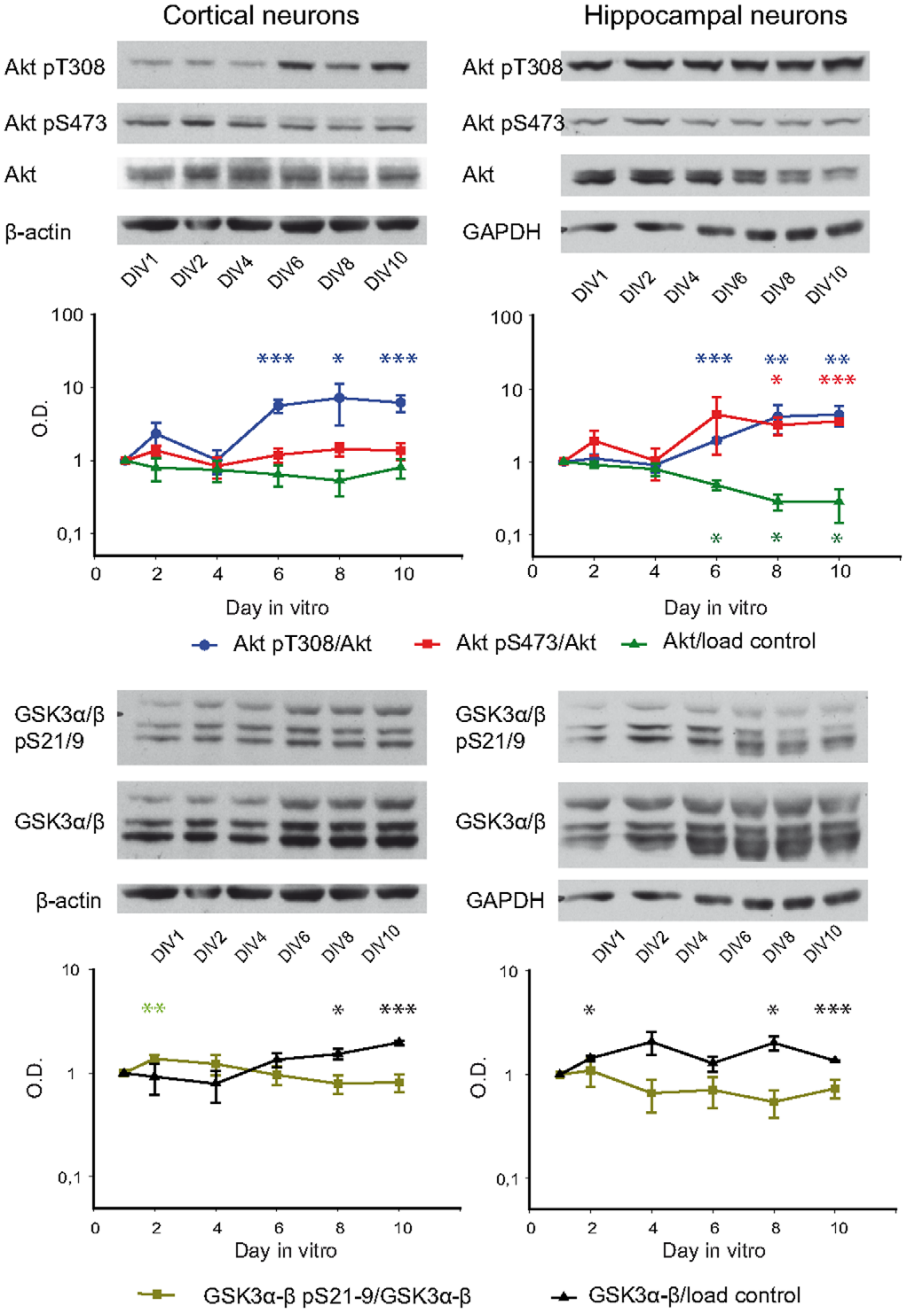
Evolution of the Akt-GSK3 pathway during neuron development. The presence of Akt and GSK3 and their phosphorylation levels were analysed in cortical (**left**) and hippocampal (**right**) neuron cultures at 1, 2, 4, 6, 8 and 10 day *in vitro* (DIV). The cell extract was analysed by Western blot using antibodies against pan-Akt, Akt pT308, Akt pS473, GSK3α/β and GSK3α/β pS21/9. β-actin was used as load control for cortical neurons and GAPDH for hippocampal neurons. Western blots were quantified and normalised with respect the control protein as indicated in Figure 1, and the values are represented in logarithmic scale. The graphs represent three independent experiments (each point represents mean ± SEM). Samples were compared to 1 DIV using Student's t test; *: *p*<0.05; **: *p*<0.01; ***: *p*<0.001. Note that Akt activating phosphorylations were increased during both cortical and hippocampal neuron development from 6 DIV on but no correlation was observed with the phosphorylation status of its substrate GSK3α/β. Total levels of GSK3α/β increased during development in both types of cultures.

Once we had obtained the temporal expression pattern of each Akt isoform and their subcellular locations, we studied the activity of Akt during neuronal development. The activity of Akt may be inferred by two different approaches. First, the phosphorylation level of Akt pT308 and Akt pS473 has been correlated with a high activity of Akt [5]. Second, the phosphorylation level of its substrate GSK3, on serine 21/9, is commonly used as a reporter of Akt kinase activity [3], [7]. Thus, we analysed our neuron cultures with phospho-specific antibodies against Akt pT308 and Akt S473 (**Figure 2**).

The analysis of cortical neurons protein extracts showed an important increase with respect to 1 DIV of total Akt pT308 levels normalized to total Akt levels which was statistically significant from 6 DIV to 10 DIV (6 DIV: 565.8±117.5%, *p*<0.001; 8 DIV: 726.4±419.1%, *p*<0.05; 10 DIV: 624,8±158.2%, *p*<0.001). However, no changes were observed in total Akt S473 phosphorylation. The analysis of total Akt showed a small non-significant reduction from 2 DIV (**Figure 2**). Interestingly, the data from hippocampal neurons cultures (**Figure 2**) showed that Akt phosphorylation at T308 and S473, increased significantly from 6 or 8 DIV with respect to 1 DIV. Akt phosphorylation levels at T308 were 195.9±14.4% at 6 DIV, *p*<0.001; 415.5±180.9% at 8 DIV, *p*<0.01; 442.5±139.1% at 10 DIV, *p*<0.001; whereas the ones for Akt pS473 were 316±88.6% at 8 DIV, *p*<0.05, and 359.2±7.52% at 10 DIV, *p*<0.001. Interestingly, in contrast to what was observed in cortical neurons, total Akt decreased significantly from 6 DIV on, its levels being 47.6±7.17% at 6 DIV, *p*<0.05; 28.3±6.64 at 8 DIV, *p*<0.05; 28±13.6% at 10 DIV, *p*<0.05, with respect to 100% at 1 DIV (**Figure 2**). Additionally, we quantified the Akt-regulated phosphorylation of GSK3 as a measure of Akt activity. Our data showed a slight rise of GSK3 phosphorylation in cortical neurons at 2 DIV and a tendency to reduction in hippocampal cultures from 4 DIV on (**Figure 2**). Total GSK3 increased significantly at 8 DIV and 10 DIV respect to 1 DIV in both cortical (8 DIV: 153.1±17.7%, *p*<0.05; 10 DIV: 197.6±9.45%, *p*<0.001), and hippocampal cultures (8 DIV: 203±31.1%, *p*<0.05; 10 DIV: 136.6±0.621%, *p*<0.001).

Next, we analysed the kinase responsible for Akt phosphorylations at T308 and S473. It is generally accepted that PDK1 and mTORC2 would be the responsible kinases for these phosphorylations [3]. Thus, considering our previous data, we analysed the amount and evolution of PDK1 and the mTORC2 component protein, mSin1, in our neuronal cell cultures.

Our data indicated that PDK1 did not show relevant differences in expression along the time in cortical or hippocampal neurons cultures, and no evident correlation was observed between the degree of Akt phosphorylation and these protein levels. (**Figure S1**). Regarding mSin1, our data indicated the presence of two isoforms; of these, only the high molecular weight isoform is part of mTORC2 [45]. When we quantified the high-molecular weigh component, we detected a low increase in hippocampal neurons and no differences when we analysed cortical ones (**Figure S1**).

### Characterization of Akt-mTORC1 signalling in neuron cultures

**Figure 3:**
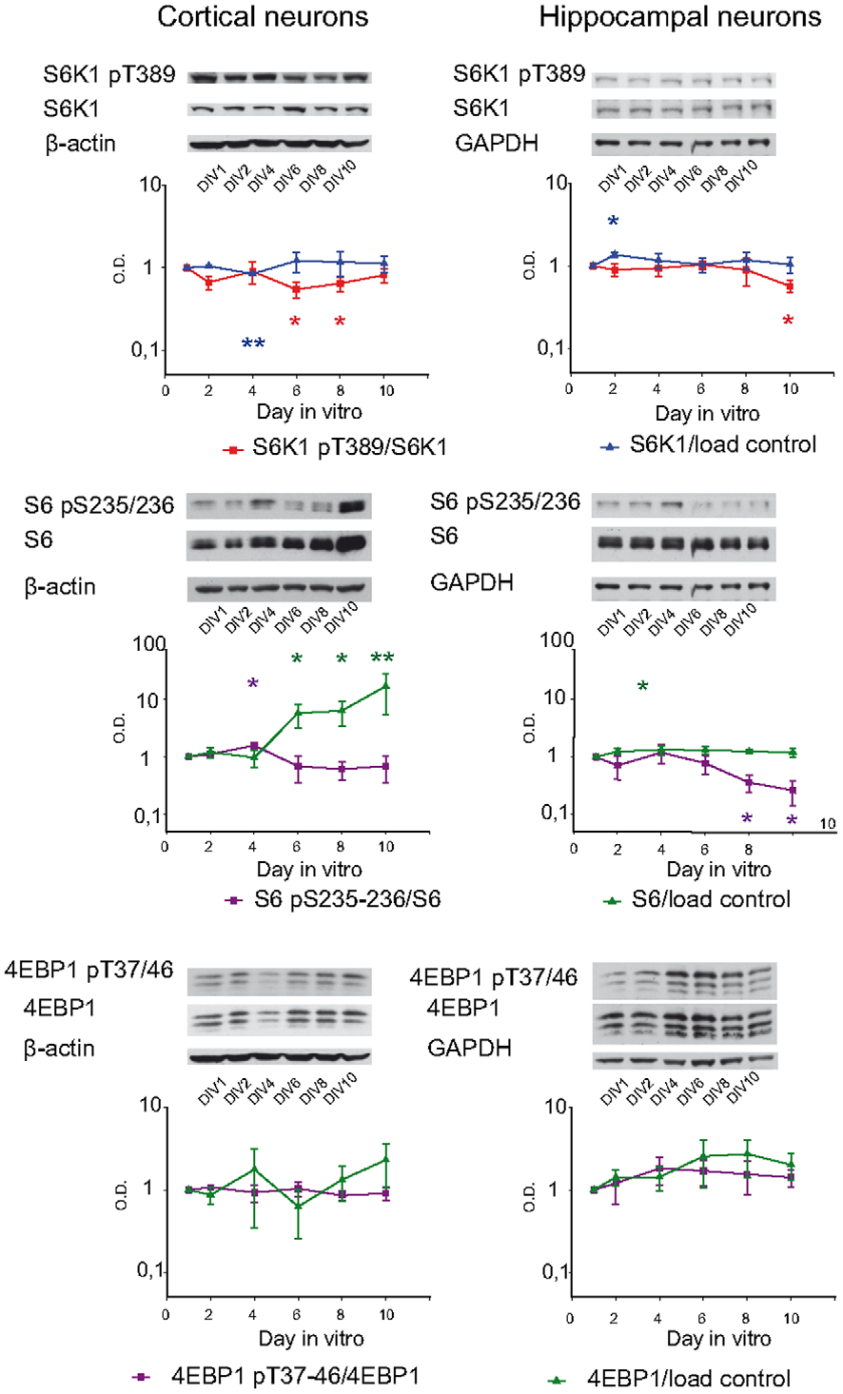
Evolution of mTORC1 substrates during neuron cultures. Elements of this pathway were analysed in cortical (*left*) and hippocampal (*right*) neuron cultures at 1, 2, 4, 6, 8 and 10 day *in vitro* (DIV). Cell extracts were evaluated using antibodies against S6K1, S6K1 pT389, S6, S6 pS235/236, 4EBP1 and 4EBP1 pT37/46. β-actin was used as load control for cortical neurons and GAPDH for hippocampal neurons. Western blots were quantified and normalised with respect the control protein as indicated in Figure 1, and the values are represented in logarithmic scale. The graphs represent three independent experiments (each point represents mean ± SEM). Samples were compared to DIV1 using Student's t test; *: *p*<0.05; **: *p*<0.01; ***: *p*<0.001. Although the mTORC1 phosphorylation on 4EBP1 remained stable in both kind of cultures, in cortical neurons S6K1 pT389 decreased on 6–8 DIV, and its substrate S6 pS235/236 showed a tendency to reduction beginning on 6 DIV. In hippocampal cultures, S6K1 pT389 was reduced on 10 DIV and its substrate S6 pS235/236 decreased on 8–10 DIV.

Besides being a substrate of Akt, mTORC1 plays an essential role in neuron development [46]. For this reason the evolution of the main elements known of this pathway were also studied (**Figure 3** **and Figure S2**).

The mTORC1 downstream proteins S6K1, its substrate S6, and 4BEP1 were analysed (**Figure 3**). In cortical neurons, there was a statistically significant reduction in S6K1 phosphorylation at T389, an activating phosphorylation regulated through mTORC1. S6K1 pT389 levels were 54.6±12.4% at 6 DIV, *p*<0.05, and 64.7±12.7% at 8 DIV, *p*<0.05 compared to a 100% at 1 DIV. Total S6K1 levels showed also faintly but statistically significant changes at 4 DIV. Phosphorylation of its substrate S6 on S235/236 was increased at 4 DIV and after this time tended to a transient decrease. Interestingly, total S6 increased from 6 DIV, being 1669.5±114.12% at 10 DIV compared to 1 DIV.

In hippocampal cultures, a decrease in S6K1 phosphorylation was also observed after 8 days of culture, which became statistically significant at 10 DIV, four days later than in cortical neurons. The total amount of both total S6K1 and S6 remained practically unaltered along the culture development, whereas the phosphorylation level of S6 decreased when compared with 1 DIV (36.8+12.3% at 8 DIV, *p*<0.05; 27.1+12.5% at 10 DIV, *p*<0.05; **Figure 3**). In this case, changes in S6K1 phosphorylation correlated with a reduction in phosphorylation of its substrate S6. Next, we analysed the phosphorylation degree of other mTORC1 pathway substrate, 4EBP1, which is phosphorylated on T37/46. Phosphorylation levels remained almost stable along the development of both cortical and hippocampal neurons.

In view of the changes observed in S6K1 and S6 phosphorylations, we tried to determine whether mTORC1 function activation by Akt was mediated through Akt phosphorylation of the mTORC1 inhibitors, TSC2 and PRAS40 [8], [9]. Our data shows that Akt-induced phosphorylation of these substrates displayed a faint but significant decrease from 2 DIV during the development of cortical neurons (**Figures S2, left panel**), that may correlate with the results observed for S6K1 pT389 and S6 pS235/236 phosphorylations, but not for 4EBP1 pT37/46. In addition, PRAS40 phosphorylation was also slightly reduced in hippocampal neurons, and it is important to note that total levels of TSC2 showed a marked decrease in the last days when compared with 1 DIV (20.5+10.1% at 8 DIV, p<0.05;: 11.4+1.72% at 10 DIV, p<0.001) (**Figures S2, right panel**).

### ShRNA-induced disruption of Akt2 and Akt3 reduces neuron viability

After the characterization of Akt and some of its upstream and downstream regulatory elements in cultured hippocampal and cortical neurons, we then examined the functions of each Akt isoform. Using interference RNA technology, we studied their roles in cell survival and axon growth, two of the main neuronal events in which Akt has been involved. Akt function has been proposed to be crucial for the maintenance of neuron viability and for neuroprotection [17], [18], [19]. Thus, we decided to study the involvement of its isoforms in cell survival.

**Figure 4:**
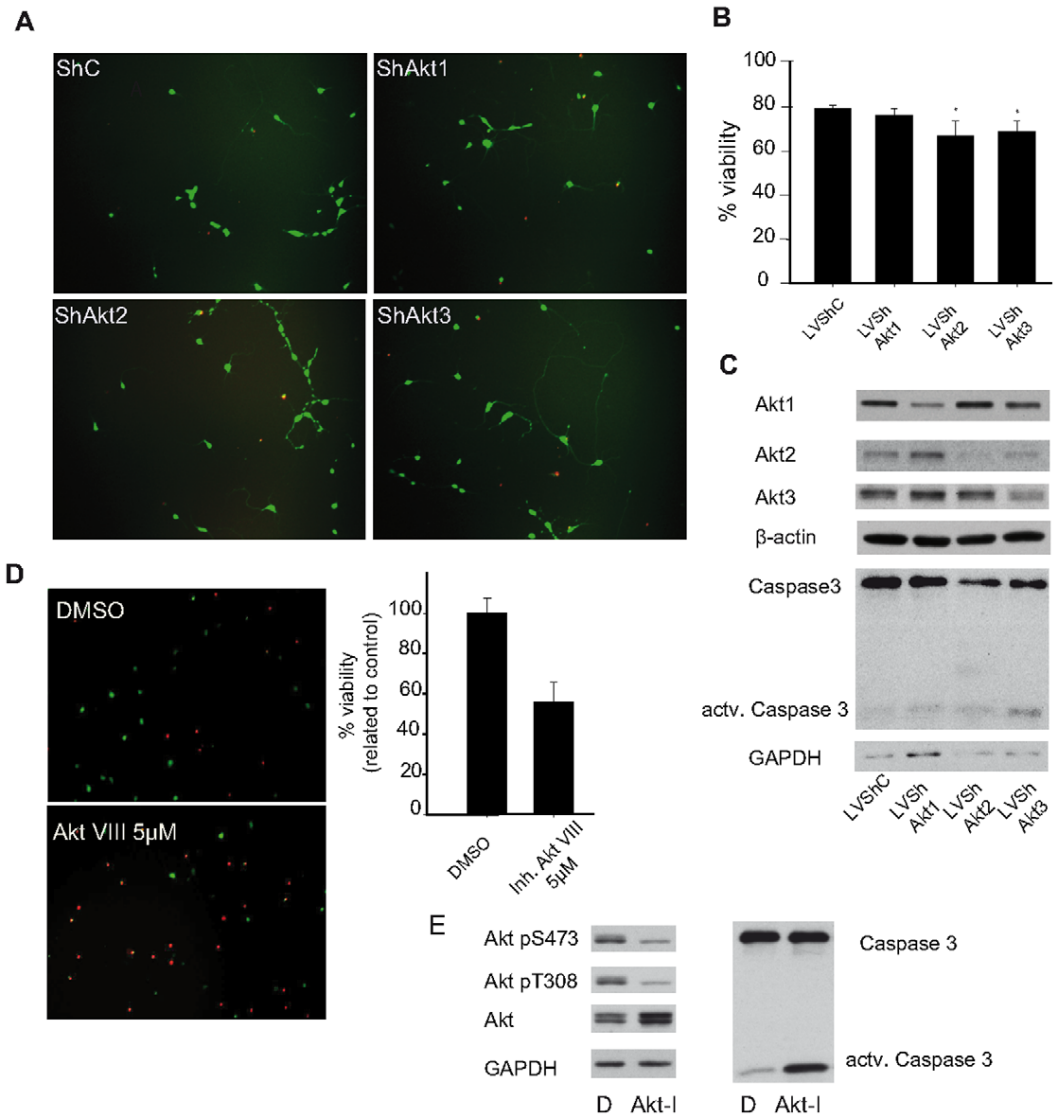
Akt isoforms differentially regulate neuron survival. Cortical neurons were infected with lentiviral vectors containing shRNAs at 1 DIV for six hours (**A–D**). As a control of the effect of total Akt inhibition, neurons were treated with the Akt inhibitor VIII 5 µM from 1 DIV (**D_E**). The viability was assayed using a propidium iodide-calcein assay performed on 4 DIV. **A.** Representative photographs for shRNA containing lentiviral infection. **B.** Percentage of viable cells (Calcein-positive). Each sample was compared to control (ShC) using Student's t test (bars represent mean ± standard deviation; *: *p*<0.05). Note that the disruption of Akt2 and Akt3 caused a significant reduction on neuron viability. **C.** ShRNA interference was confirmed at 4 DIV by inhibition of protein expression using specific antibodies. Apoptosis was evaluated at 4 DIV by the presence of active **caspase 3** associated to shRNA-induced Akt2 and Akt3 reduction (*Lower panel*). **D.** To identify the maximal effect due to Akt inhibition, in parallel we treated neurons with for Akt inhibitor VIII (5 µ m). Representative photographs for Akt inhibitor VIII treatments. The graphs correspond to the quantitative determination of viable neurons, either after inhibitor VIII or DMSO treatments (represented as percentage of calcein-positive cell). In each independent experiment (n=3), data from Akt-VIII was compared to control (DMSO) using Student's t test (bars represent mean ± standard deviation; **: *p*<0.01). **E.** Inhibition of Akt was confirmed at 4 DIV by reduction of Akt activating phosphorylation levels (Akt pT308 and Akt pS473). And the maximal level of apoptosis was evaluated at 4 DIV (*right panel*), by the presence of active caspase 3 associated to Akt-VIII inhibitor (**Akt-I**).

Using specific shRNAs, we analysed neuronal cell viability after removal of each isoform on cortical neurons (**Figure 4**). Cortical neurons were infected with lentiviral vectors at 1 DIV, once the neurons have adhered to the plate and after a day of adaption to culture medium, and cell viability was determined three days after infection using a propidium iodide-calcein assay (**Figure 4A–B**). At this time, the efficiency of shRNA interference was established by Western blot. Results showed a reduction of over 50% for every isoform in all cases (**Figure 4B**). Our data indicates that only the disruption of Akt2 and Akt3 led to a slight but statistically significant reduction of neuron survival percentage under the standard culture conditions when compared with control scrambled shRNA infected neurons (79.04±1.74% in shControl:; 60.60±6.58 in shAkt2, *p*<0.05, 70.37±4.76% in shAkt3,, *p*<0.05, n=3) (**Figure 4B–C**). This reduction on cell viability correlated with a tendency of an increase in active caspase-3 (the most commonly used apoptotic marker [47]), in either Akt2 and Akt3 (**Figure 4C**).

As a positive control, the apoptotic effect of Akt inactivation was confirmed using the specific Akt inhibitor VIII, which binds to Akt and impedes its phosphorylation by PDK1 and mTORC2 [48]. The propidium iodide-calcein assay showed that inhibition of Akt induced a marked decrease in neuron viability and only 55,4±10,1% of neurons were viable (**Figure 4D**). The inactivation of Akt was confirmed by the decreased phosphorylation of both T308 and S473 Akt residues (**Figure 4D–E**). As expected, this inhibition induced a high increase of cleaved caspase 3 (**Figure 4E**). These results suggest that Akt2 and Akt3 play specific and non-redundant roles in neuron survival by regulating apoptotic pathways.

### Role of Akt isoforms in axon development

**Figure 5:**
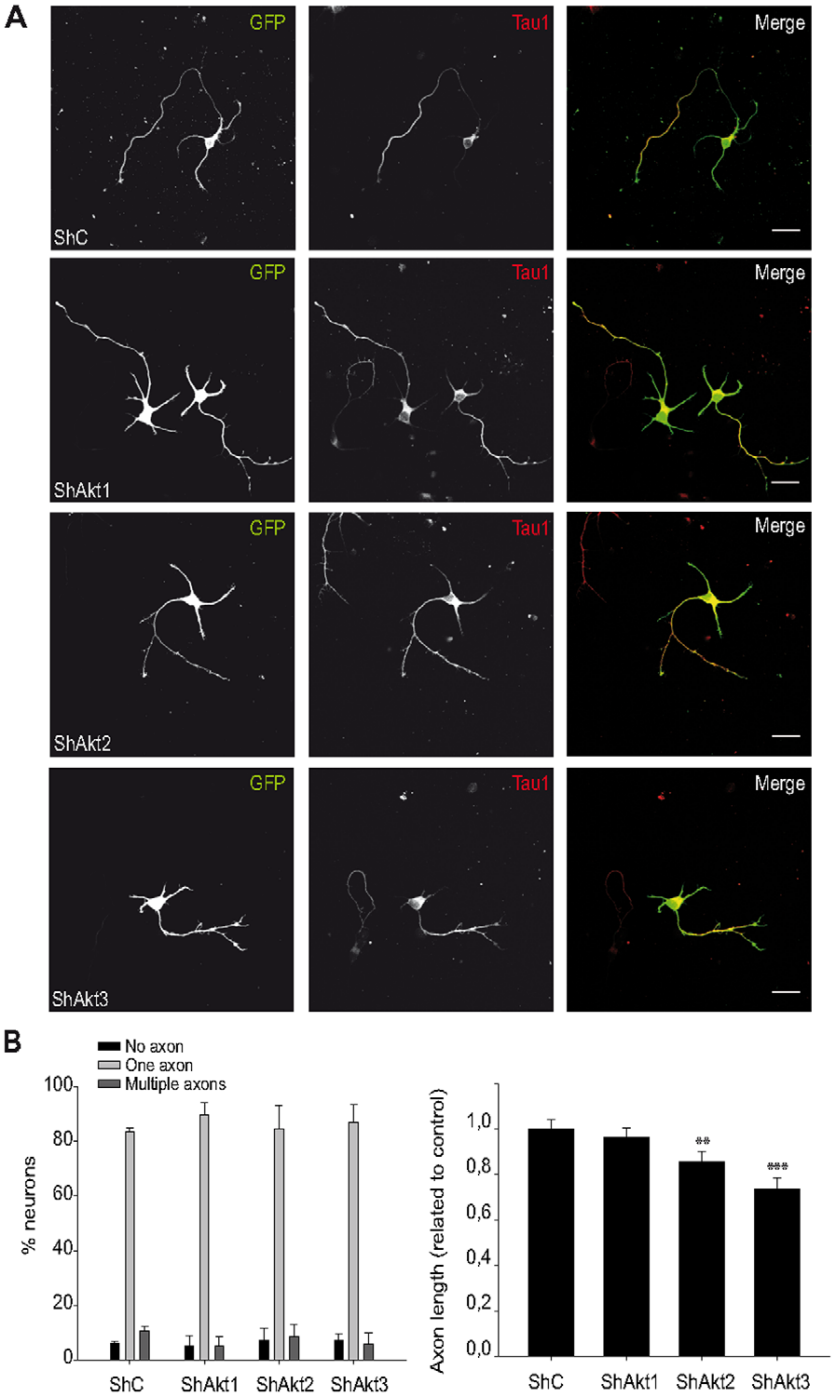
Akt isoforms differentially regulate axon growth but do not affect axon establishment. Hippocampal neurons were nucleofected with the indicated shRNAs and a GFP-expressing plasmid, and analysed later on DIV 3. **A.** Neurons were fixed and anti-Tau-1 antibody was used as an axonal marker. The photographs correspond to a representative field for each experimental condition in which the Tau-1 positive and/or GFP-positive neurons were observed. Scale bar: 25 µm. **B.** Data indicated that Akt isoforms disruption did not induce a statistically significant change in neuron polarity (each condition was compared to control using Student's t test; bars represent mean ± standard deviation). **C.** However, when the axonal length were determined (form those GFP-positive and Tau-1-positve neurons), the disruption of both Akt2 and Akt3 caused a reduction of axon length (samples were compared to control using Mann-Withney test as they did not show a normal distribution; bars represent mean ± SEM; **: *p*<0.01, ***: *p*<0.001).

Axon growth is one the first and more characteristic events of neuron development and it is regulated by a complex network of signaling pathways that involves extracellular and intracellular molecules. It is generally accepted that the PI3K pathway plays a critical role in promoting both axon differentiation and growth. Consequently, we asked if Akt isoforms are differentially involved in this cellular process. For this purpose, we used hippocampal neurons as the most extended model to study axonal polarity [44]. In standard hippocampal cultures, an axon is established between 1 DIV and 3 DIV [49]. Therefore, the isoform-specific shRNAs were nucleofected into hippocampal neurons before plating, to obtain a highly efficient delivery of shRNA-containing plasmids. Next, neurons were grown under the standard conditions for 3 days, fixed, and axon growth was checked by immunocytochemistry with the specific axonal marker Tau-1 [50] (**Figure 5A**). The presence of axons and their length were quantified in neurons lacking each Akt isoform.

Despite the effects described for whole Akt activity inhibition [24], [25], [26], [27], [28], depletion of each Akt isoform did not exert statistically significant changes in neuron polarity and more than 80% of neurons extend one axon (**Figure 5B**). However, interference of Akt2, and to a greater extent that of Akt3, reduced axon length with respect to control neurons. Axonal length of shAkt2 nucleofected neurons was only 85.7±4.26% of that shown by control neurons (*p*<0.01), and 73.8±4.73% in the case of shAkt3 (*p*<0.001) (**Figure 5C**).

### Akt isoforms regulation of cell signaling

**Figure 6:**
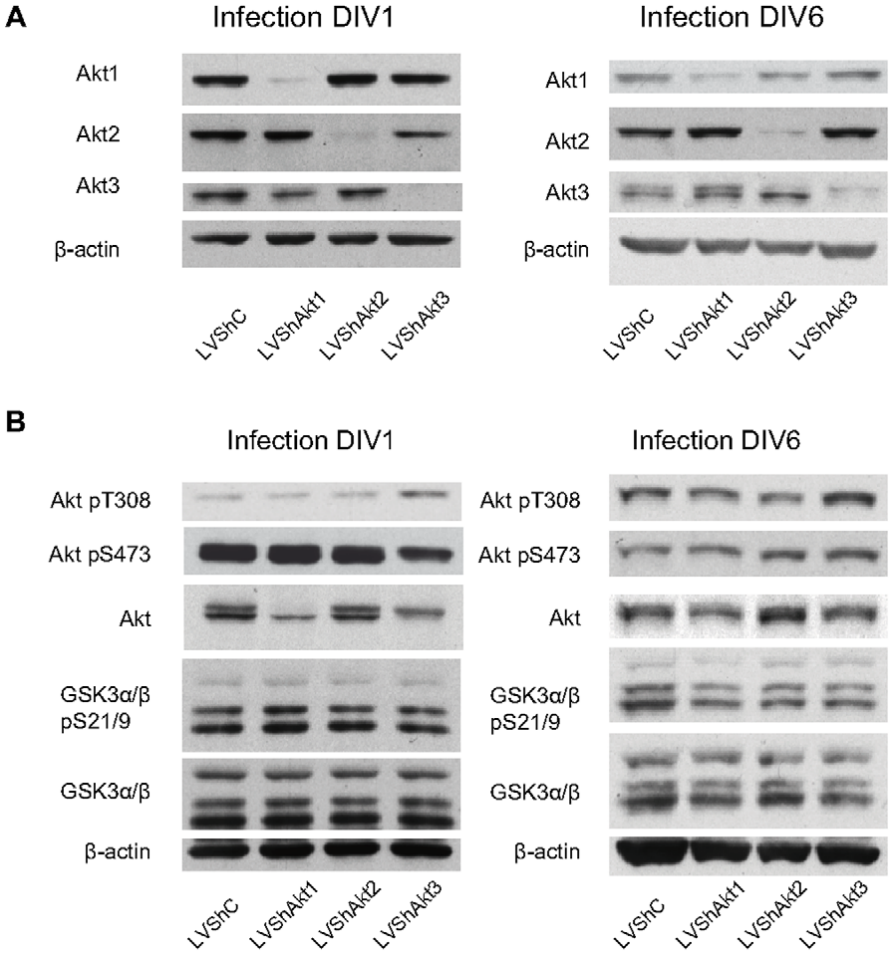
Regulation of Akt-GSK3 pathway by Akt isoforms. Cortical neurons were infected with lentiviral vectors containing shRNA at two times (1 DIV and 6 DIV) for six hours. 72 h after the infection cell extracts were obtained (at 4 DIV and 9 DIV respectively). **A.** As an internal control, shRNA induced interference was confirmed by protein expression using specific antibodies against each Akt isoform. **B.** The effect of Akt interference was analysed using antibodies against Akt and GSK3: pan-Akt, Akt pT308, Akt pS473, GSK3α/β and GSK3α/β pS21/9. β-actin was used as load control. Akt1 and Akt3 may be identified as two different bands marked by the pan-Akt antibody. Although Akt2 disruption caused a slight decrease in Akt pT308, no other significant changes are observed.

Our results show that specific Akt isoforms disruption have differential effect on neuron viability and axon growth, thus we decided to analyse the effect of Akt isoforms suppression on downstream elements. Considering that Akt isoform levels changed along the culture development (**Figure 1**), neuronal cultures were infected at two significant times, 1 DIV and 6 DIV. After lentiviral transduction, neurons were harvested 3 days later to study the potential variability on signaling. Following the same previous scheme used in our study along neuronal development, Akt activating phosphorylation and Akt substrates were analysed. The efficiency of interference was determined by Western blot as an internal control after each experimental condition, using the specific antibody against each Akt isoform (**Figure 6A**).

The interference of Akt1 and Akt3 did not modify the level of Akt pT308 significantly, even though Akt1 represents half of the total Akt expression (**Figure 6B**). In contrast, Akt2 disruption was related to a reduction of total Akt pT308 both in 1 DIV infection (62.29%±8.80 respect to the control; *p*<0.01, n=3) and 6 DIV infection conditions (52.41%+7.20 respect to the control; *p*<0.01, n=3), although no significant changes were observed in total Akt pS473 (**Figure 6B**). The use of Pan-Akt antibody did result in a western-blot double band. Akt1 interference is linked to a reduction in the upper band and Akt3 disruption is linked to a decrease in the lower one (**Figure 6B**). Despite the changes in Akt isoforms expression and Akt phosphorylation at T308, when we analysed GSK3, one of the main substrates of Akt, we did not observe any significant changes in either GSK3 phosphorylation or total expression levels (**Figure 6B**).

**Figure 7:**
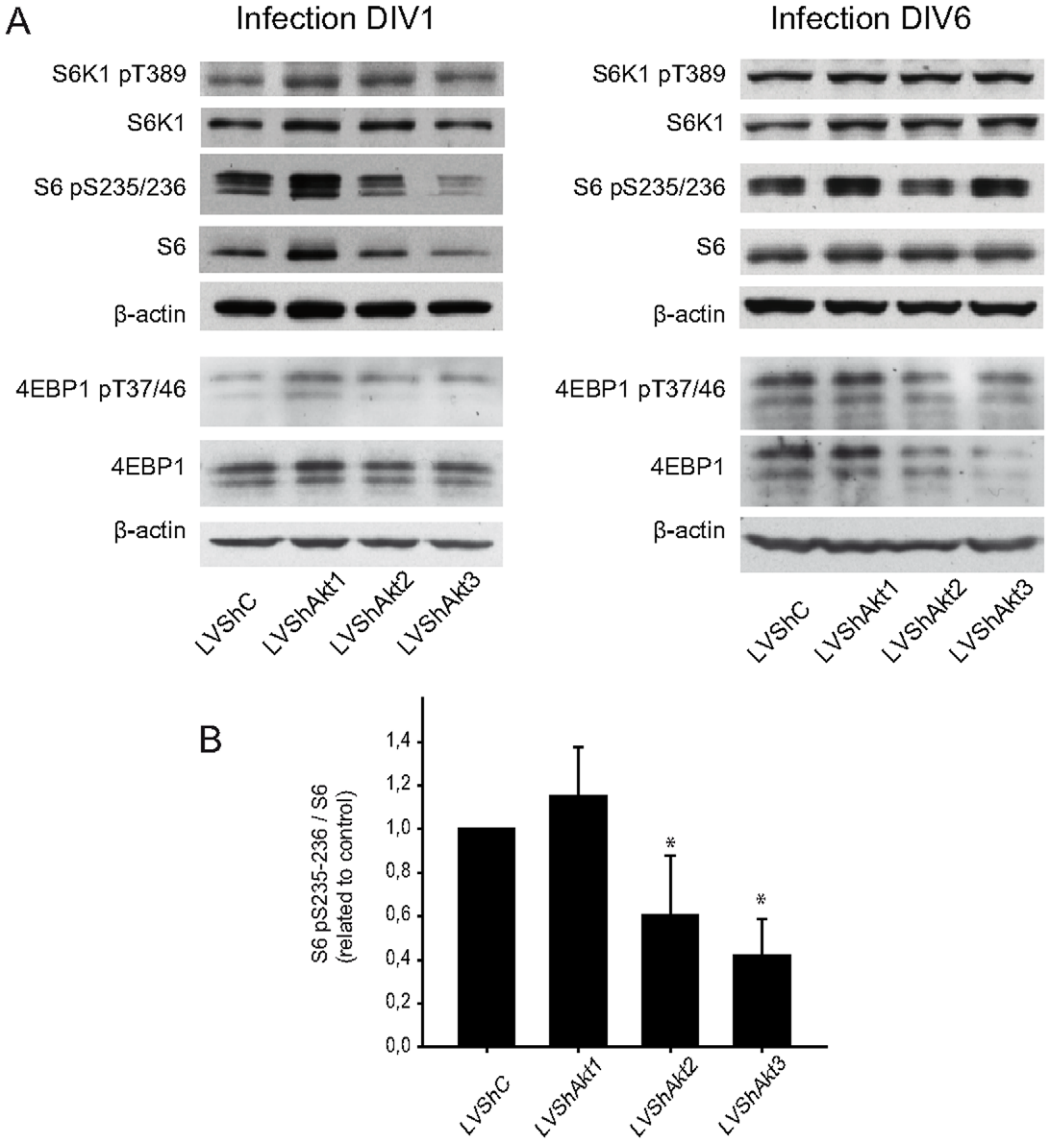
Regulation of mTORC1 substrates by Akt isoforms. Cortical neurons were infected with lentiviral vectors containing shRNA at two times (1 DIV and 6 DIV) for six hours; proteins were extracted 72 h after the infection (at 4 DIV and 9 DIV respectively). The cell extract was examined using antibodies against the canonical substrates of mTORC1 pathway: p70 S6K1, p70 S6K1 pT389, S6, S6 pS235/236, 4EBP1 and 4EBP1 pT37/46. β-actin was used as load control. The interference was confirmed by protein expression using specific antibodies as previously indicated (see **Figure 6A**). **A.** Note that Akt2 and Akt3 regulated S6 phosphorylation but no statistical change was observed in the direct mTORC1 substrates S6K1 and 4EBP1. **B.** The relative level S6 pS235/236 with respect to the total S6 was determined, at 4 DIV by Western blot (each condition was compared to control using Student's t test; bars represent mean ± standard deviation).

Then, we analysed the mTORC1 pathway, the main effector of Akt in cell growth and translation control (**Figure 3** and **Figure 7**). First, we analysed the mTORC1 regulators, TCS2 and PRAS40. Our data showed that after Akt-isoform interference no statistical change was obtained in TSC2 and PRAS40 (**Figure S3**). Next, we analysed the phosphorylation state of S6K1 on T389, but interestingly it was almost not modified after infection with shAkts, in either 1 DIV or 6 DIV neurons (**Figure 7**). However, the analysis of S6 showed that S6 phosphorylation was reduced by shAkt2 when infecting at 1 DIV (60.60±27.04% respect to the control; *p*<0.05, n=3) and 6 DIV (48.52±22.96%; *p*<0.05, n=3), and more severely reduced by shAkt3 when infecting at 1 DIV (41.99±16.67%; *p*<0.05, n=3). In contrast, no change was detected after shAkt1 infection. When we analysed the other mTORC1 direct substrate, 4EBP1, no statistical change was observed in its T37/46 phosphorylation (**Figure 7 A**).

Finally, it has been proposed that Akt isoforms could regulate upstream events in the MAPK pathway in different cell types [55], [56]. Therefore, we asked if Akt isoforms could regulate the MAPK pathways. We checked the final activating phosphorylation of the three main MAPKs (ERK, JNK and p38), commonly used as a reporter of the activation of these pathways [51]. No statistically significant variation was found in either of them in standard neuron culture conditions (**Figure S4**).

## Discussion

### Akt isoforms along neuronal development

We have analysed the roles of Akt isoforms during development of cortical and hippocampal neuron cultures. We first observed variations in specific Akt isoform expression levels and in some of their substrates during development. Our results show that the ratio and the timing of expression of each isoform is different, which is very evident for the reduction of Akt2 expression levels in cortical neurons. Even though the specific commercially available antibodies did not permit the immunocytochemistry analysis of the neuronal location of each isoform, the subcellular distribution obtained after cell fractionation allowed us to propose that Akt3 presents a higher nucleus to cytosol ratio.

In general, the decrease in Akt isoforms contrasts with the increase in Akt activity, which was inferred from the levels of the activating phosphorylations, in both types of neurons. However, when we analysed some direct substrates such as GSK3 or S6K, GSK3 serine phosphorylation showed only a faint increase whereas S6K1 phosphorylation on threonine 389 appeared to be unmodified in cortical neurons, and showed a minor reduction in hippocampal neurons. Importantly, when we analysed the total S6 protein levels an important increase was detected in cortical neurons, but the S6 phosphorylation decreased in both neuronal models with respect to the S6 total protein. Thus, the increase in Akt phosphorylation after Akt-interference may be a compensatory mechanism, trying to maintain TORC1 activity and GSK3-inhibition.

In general, the decrease in Akt isoforms contrasts with the increase of activating phosphorylations of total Akt, in both types of neurons. However, when we analysed some substrates of this pathway, little correlation was found between these phosphorylations and downstream substrates, such as GSK3 or the mTORC1 pathway. GSK3 serine phosphorylation, which is commonly used as a reporter of Akt activity, showed only a faint increase. Nevertheless, the total GSK3 was also increased, so the higher activation of Akt could be compensating the greater amounts of this substrate; thus, GSK3 inhibition could be maintained at suitable levels for correct cellular function.

Conversely, S6K1 phosphorylation on threonine 389, which is mediated by mTORC1 appeared to be unmodified in cortical neurons, and showed a minor reduction in hippocampal neurons. Importantly, when we analysed the total S6 protein levels a significant increase was detected in cortical neurons, but the S6 phosphorylation decreased in both neuronal models with respect to the S6 total protein. S6 phosphorylation mediated by the S6Ks has been traditionally linked to cell growth [41], [52], so the reduction observed could reflect a decrease in the growth rate during development. However, the phosphorylation of 4EBP1, also directly regulated by mTORC1, showed no significant changes during neuron culture development. As it will be discussed later, S6K1 activity is also regulated by other proteins besides mTORC1 [53], so the difference observed between S6K1>S6 and 4EBP1 might be due to a regulation of S6K1 independent of mTORC1.

### Functional redundancy and divergence of Akt isoforms

The contrast between Akt isoform expression and GSK3 or S6K1 activities observed during neuronal development points to either a functional redundancy or a functional divergence of Akt isoforms that may mask the specific function of each isoform. Our results show that suppression of one specific Akt isoform results in faint changes in most of the Akt downstream substrates during neuronal development. However, when we analysed the effect of each one of the Akt shRNAs on neuron survival and axon development, only Akt2 and Akt3 disruption induced a little but significant reduction of neuronal survival and axonal length. Thus, as a perfect example of paralogs, Akt isoforms show high functional redundancy and some evidence of function divergence. In fact, Akt1 suppression may be compensated by Akt2 and/or Akt3 expression, but neither Akt2 nor Akt3 loss of function is completely compensated by Akt1 expression.

Although we have focused on the effect of Akt isoforms in differentiated neurons, our results are consistent with previous studies regarding the global effect of these proteins. Knock-out mice for each isoform are viable and show only minor alterations [4]. However, total Akt is believed to play a central role in the nervous system, as many studies based on pharmacological inhibition or gain of function Akt mutants have been proposed to be crucial for neuron survival and growth [17], [18], [19] or axon establishment [24], [25], [26], [27], [28] among many other processes. Altogether, both our results and previous data suggest that Akt isoforms show substantial compensation in many processes.

However, single isoform knockout experiments have also reported specific functions for these proteins. For example, Akt1 plays a role in placental development, growth and adipose tissue, Akt2 is related to growth and insulin response and Akt3 is linked to brain growth and oligodendrocyte genesis [4]. They also show a differential tissue distribution, which may be related to differential function [11] and are amplified in different cancer types [10]. Differential regulation of downstream substrates under different conditions has also been reported for distinct cell types [54], [55]. Consistent with these observations, we have found that the isoforms of Akt differentially regulate some aspects of neuronal function.

Most of the work describing specific functions of Akt isoforms indicate that Akt1 and Akt2 differentially regulate many cellular processes, sometimes even playing opposites roles [54]. These isoforms have been extensively studied in regulation of metabolism [10], [56] and cancer [10], [54]. So far, Akt3 has not been as deeply characterized as its two paralogs. Interestingly, our results show that Akt3 disruption exerts the most prominent effect on differentiated neurons rather than Akt1 or Akt2. This effect could be specific for the cell type we have studied, as Akt3 is restrictively distributed, with a high grade of expression in the nervous system [11], while Akt1 and Akt2 are almost ubiquitously expressed in all tissues.. In summary, our results suggest that Akt3, and Akt2 in a minor manner, might play a role in some aspects of neuron biology that could be specific for this cell type.

### Akt isoforms differentially regulate neuron viability

We analysed cell survival processes in which Akt has been involved. Different elements under the control of PI3K have been particularly associated with the maintenance of neuron viability and in neuroprotective mechanisms [17], [18], [19]. In fact, it is generally accepted that class I PI3Ks play a central role in cell survival in many if not all cell types [57]. However, our data indicated that only the disruption of Akt2 and Akt3 induces a small but significant reduction of cell viability in cortical neurons that correlates with the activation of caspase 3 [58]. These results suggest that some aspects of Akt regulation of apoptotic pathways are exclusive to Akt2 or Akt3 despite some apparent redundancy among the isoforms.

Many works show that Akt is related to the regulation of apoptosis by a complex cell signaling network; the elements of this network may be different in distinct cellular systems and show a precise subcellular distribution, being located in the cytosol, nucleus or mitochondria (reviewed in [59]). GSK3 is one of the main substrates of Akt and its inhibition has been proposed as an essential mechanism for the maintenance viability of neuronal cells [17]. However, our data showed no change in GSK3 phosphorylation associated with decreased cell viability induced by Akt2 and Akt3 disruption. Thus, we conclude that the ablation of a single isoform is not directly linked to the classical inhibition of GSK3 pro-apoptotic activity.

Although we have not yet determined the downstream molecular mechanism, our results suggest that, at least in some neuronal types, one of these isoforms would control neuronal survival. Interestingly, the Akt3 knockout experiments show results similar to ours, as hippocampal cultured neurons from Akt3^−/−^ mice show reduced cell viability in response to injury but no reduction was observed in tissue for GSK3 phosphorylation [16]. Akt3 has been localized in the mitochondria in HEK293 cells [60] and our data indicate that it presents a higher nuclear expression than any other Akt isoform. As mentioned above, some substrates of Akt involved in the regulation of cell survival are located in these two subcellular structures [59]. Thus, Akt3 could be involved in fine regulation of apoptotic mechanisms in these subcellular compartments. Besides the classical action of GSK3 in neurons, in other cell types many proteins have been proposed to be associated with the control of the apoptosis /survival pathway mediated by Akt [59], such as hexokinases (regulators of the cytochrome C in mitochondria) [61], Bad [62], [63] or Bax [64], pro-apoptotic and anti-apoptotic transcription factors, such as FoxO [65], [66], CREB [67] or Mdm2>p53 [68]. Further work will help us to elucidate the specific function of Akt isoforms in this complex process.

### Role of Akt isoforms in axon growth

It has been proposed that Akt plays a critical role in axon development [24], [25], [26], [27], [28] partially based on the observation that PI3K [69], as well as IGFR-I, have been reported as master regulators of axonal polarity [70]. In addition Akt has been proposed to be a pivotal element in this process because PI3K downstream elements such as GSK3 play an essential role in axonal elongation [24], [25], [26], [27], [28], [71], and lack of GSK3α or GSK3β impede axonal elongation [71]. Neuronal polarization is the basis of neuronal morphological differentiation and it is crucial for correct neuron physiology [44], [72]. Thus, we decided to evaluate the specific function of each isoform in the most extended model for these studies in central nervous system cells: cultured hippocampal neurons. We focused on two parameters that reflect different cell processes: axon establishment, which is a marker of cell polarization and asymmetric distribution of cell components, and axonal elongation. In hippocampal cultures, embryonic neurons start to project similar neurites a few hours after plating, one of which grows at a faster rate than the others and becomes a differentiated axon at 2–3 DIV by a molecular mechanism not completely understood [49]. However, our results indicate that Akt isoform depletion does not affect axonal polarization, even though the final length of the axon is statistically reduced. This lack of effect may reflect the lack of GSK3 phosphorylation changes. Also, the data suggest that compensatory mechanisms can resolve the possible defects produced by suppression of single Akt isoforms. In addition, GSK3 has proven to be inhibited by other AGC kinases under certain conditions (PKC, S6K, SGK, etc.) [7], and thus, the establishment of neuronal polarity and axon differentiation may be driven even by other proteins. In fact, some βββevidence suggests that atypical PKC could be the former GSK3 regulator involved in neuron polarity rather than Akt [73].

The reduction of axonal length opens the possibility that others Akt substrates may be implicated in this process. Indeed, mTORC1 has been proposed as another essential factor in axon formation [37], [38], [74], [75]. However, to our knowledge no work has directly established a link between Akt regulation of mTORC1 and axon formation, so Akt may not participate in mTORC1 control of polarization. Two elements of this pathway have been proposed to be critical: 4EBP1 [37] and S6K1 [38]. In cortical neurons, the first one is not altered by single Akt isoform disruption, and mTORC1-regulated S6K1 phosphorylation shows only minor changes, which correspond with the unaltered neuron polarity we observed for hippocampal cultures. These results suggest that both Akt2 and Akt3 could be related to mTORC1-regulated cell growth, which has been related to S6 phosphorylation [41], but no isoform seems to be indispensable for mTORC1 regulated cap-dependent translation initiation, which is mediated through 4EBP1 [76].

### Differential regulation of mTORC1 substrates implicated in cell growth by Akt isoforms

ShRNAs against Akt2 and Akt3 have a significant effect on hippocampal axon length, and there is a correlation between this effect and a reduction in S6 phosphorylation in cortical neurons at the times of maximal axon growth which were considerably clear when Akt3 was depleted. Axon development is the main event in general neuron growth [49] and the S6K>S6 pathway is considered one of the master regulators of cell growth [41], [52]. Thus, Akt2 and Akt3 isoforms may be regulating general neuron growth through the S6K>S6 pathway at this stage and the observed reduction in axon length may reflect this global process (cell growth) instead of an effect specific for axon development.

Knock-in experiments indicate that S6 phosphorylation on S235/236 is strongly associated with cell growth [52]. However, we did not observe a statistically significant reduction of S6K1 pT389, with poor correlation with S6 phosphorylation. Although S6 phosphorylation is commonly used as the activation reporter for this pathway, the S6Ks are a node of convergence for distinct cell signaling pathways [53], including the mTORC1, PDK1 [77], and some Ser-Pro directed kinases that may be identified as CDKs [78], [79]. Also, there are two S6 kinase paralogs, which are believed to be similarly regulated: S6K1 and S6K2 [80]. S6K2 is ubiquitously expressed and seems to have a crucial role in this pathway [80]. Indeed, some data suggest that S6K2 may be the main kinase responsible for S6 phosphorylation, rather than S6K1 [81], [82]. The PI3K pathway has been shown to play a central role in its regulation [83]. S6K1 and S6K2 show different subcellular distribution, as S6K1 is believed to be primary cytosolic and S6K2 is located in the nucleus [80], suggesting that both are implicated in different cellular processes. Unfortunately, we lack of the tools necessary to study S6K2 yet, as it has received less attention than S6K1 during the last years.

The regulation of S6Ks by Akt isoforms is not clear. Our results indicate that the direct substrates of mTORC1, 4EBP1 and S6K1 are not altered by Akt2 and Akt3 disruption, but the S6K activity towards S6 is reduced. We have observed a similar “uncouple effect" between mTORC1 action on its direct substrates and S6 phosphorylation during cortical and hippocampal culture development. There are different explanations for this observation. Akt isoforms might play a role in the regulation of S6K affinity towards its S6 substrate (i.e., through modification of scaffolding proteins or cellular distribution), could also regulate S6K activity in an mTORC1 independent manner, or even may act upstream of a mTORC1 pool specifically related to S6 phosphorylation, possibly by regulation of S6K2 activity. The fact that both Akt3 and S6K2 show nuclear distribution seems to be particularly attractive for future studies.

Interestingly, our results on Akt3 are similar to the ones on brains of Akt3 knock-out mice [15], [16]. In both cases, a reduction in growth was associated with a marked decrease in S6 phosphorylation, but minor changes or no alteration in S6K1 were reported. However, we observed a S6 phosphorylation recovery on 9 DIV for Akt3 disrupted cortical neurons, although this difference could be explained by temporal changes during culture (the fact that total S6 undergoes a marked increase at these days in culture is a point worthy to mention). In agreement with our results, Akt1−/− mouse brains show no statistically significant reduction in cell size or S6 phosphorylation [15]. Thus, Akt3 seems to be a master regulator of differentiated neuron growth. Indeed, some publications suggest that Akt3 may be responsible for rare cases of human microcephalia associated with corpus callosum abnormalities caused by cytogenetic deletion of 1q44-qter, which contains the human Akt3 gene [84], [85], [86]. This phenotype strongly resembles the one of Akt3−/− mice, so the study of Akt3 may help in the understanding of the molecular basis of human pathologies.

Another complementary research line that linked class I PI3K>Akt pathway to neuron growth is based on studies on PTEN depletion. PTEN is the main physiological class I PI3K>Akt pathway inhibitor, a lipid phosphatase that dephosphorylates phosphatidylinositol (3,4,5)-triphosphate (PIP_3_) to phosphatidylinositol (4,5)-biphosphate (PIP_2_) and therefore deactivates class I PI3K induced signalling [87]. In fact, brains of conditional PTEN knock-out mice show the inverse phenotype of Akt3−/− mice: neuronal hypertrophy kinked to a marked increase in S6 phosphorylation and reversed by mTORC1 inhibition [34], [88], [89]. In humans, PTEN inactivating mutations have been linked to neuronal diseases, including autism [90] or Lhermitte-Duclos disease, which is in fact characterized by hypertrophy of the *stratum granulosum* of the cerebellum due to a deregulation of postnatal neuron growth [91]. Our results may help to explain the molecular mechanisms controlling these syndromes. On the basis of our results and previous work from other laboratories, we believe that a study combining Akt3 depletion and PTEN inactivation in neuronal growth and cell growth related pathways would be very informative and could shed light on the roles these biochemical pathways play in neurological diseases.

In summary, our data show that the three Akt isoforms show redundancy of function but suggest a fine regulation of some aspects of apoptotic regulation and axon growth. Future work will help to elucidate the complex molecular mechanisms underlying these processes and the fascinating relation between Akt paralogs and the cell growth machinery. Our results may also help to develop a comprehensive model of the molecular basis of syndromes related to neuron growth.

## Materials and Methods

### Cells and cell culture

HEK293T (ATCC® Number: CRL-11268) human embryonic kidney cells were used to package viral particles generated to infect neurons. This cell line was maintained in Dulbecco's modified eagle medium (DMEM) with 10% fetal bovine serum (FBS), 2 mM glutamine and Penicilin/Streptomycin at 37°C and 5% CO_2_.

Hippocampal and cortical neurons were obtained from E18 mouse embryos as previously described [43]. Briefly, after isolating the embryonic hippocampi or cortex from 8–10 mice in Ca^2+^-and Mg^2+^-free Hanks Buffer Salt Solution (HBSS 1×, GIBCO), hippocampi were incubated in 0.25% trypsin (GIBCO) for 15 min at 37°C. 1 mg/ml DNAse-I (Roche) was also added in the case of cortex. Trypsin was eliminated by washing three times with Hank's buffered salt solution (HBSS) and tissue was homogenized using two Pasteur pipettes. Cells were counted and plated in 1 mg/ml poly-lysine (Sigma) coated dishes containing plating medium (MEM, 20% Glucose, 10% horse serum–GIBCO- and antibiotics), and incubated for 3 h. Afterwards, the medium was changed to neurobasal medium supplemented with B27 (GIBCO). Cell density was 4×10^4^ cells/cm^2^ for Western blot analysis, and 10^4^ cells/cm^2^ for immunocytochemistry. Neurons were maintained under these conditions for the time indicated in each experiment.

### Plasmids

ShRNA containing pLKO1.puro plasmids were from MISSION® (Sigma): TRCN0000022937 for Akt1, TRCN0000055260 for Akt2 and TRCN0000054726 for Akt3. The scrambled control shRNA was from Addgene (#1864, [92]); pEGFP.N1 was from Clontech. pM2D.G and pCMVΔR8.2 plasmids were used for lentiviral packaging [93].

### Lentiviral vector packaging and infection

Lentiviral vectors were packaged in HEK-293T cells as previously described [93]: pM2D.G, pCMVΔR8.2 and shRNA containing pLKO1.puro were transfected into HEK-293T in a ratio 1∶1∶3. 48 hours after transfection medium containing the viral particles was filtered using 0.45 µm low binding protein filters. Cortical neuron cultures were infected with this medium for six hours at 37°C .

### Transfection

Hippocampal neurons were nucleofected with the Amaxa Basic Nucleofector Kit for Primary Mammalian Neural cells (Lonza, Basel, Switzerland); a total of 3 µg of plasmid plus 1 µg pEGFP.N1 were introduced into 3×10^6^ cells. Transient transfection of N2a cells was driven by LipofectAMINE 2000 (Invitrogen) in OPTIMEM medium (GIBCO) at a ratio of 1 µg DNA per 3 µl of LipofectAMINE 2000 for 5×10^5^ cells.

### Western Blot analysis

Cell extracts were prepared in lysis buffer containing 200 mM HEPES at pH 7.4, 100 mM NaCl, 100 mM NaF, 1 mM Na_3_VO_4_, 5 mM EDTA, 1% Triton-X-100 and a protease inhibitor cocktail (COMPLETE™, Roche, Basel, Switzerland). Cells were left for 20 minutes on ice in lysis buffer. After this time we obtained the cell extract and we added Laemmli sample buffer [94]. Samples were boiled for 10 min and resolved by Tris/Glycine SDS-Polyacrylamide gel electrophoresis. Proteins were transferred for 145 minutes at 0.35A to a nitrocellulose membrane (Whatman, Dassel, Germany) in the presence of 20% methanol. Non-specific signals were blocked by incubating the membrane in phosphate buffered saline (PBS)-Tween 20 (PBT) containing 7.5% non-fat milk for 10 minutes. Primary and secondary antibodies were incubated in PBT. Western blots were analysed with GS-710 densitometer (Biorad, Hercules, California).

### Subcellular fractionation

Cells were harvested in 320 mM sucrose, 20 mM HEPES pH 7.4, 10 mM KCl, 1.5 mM MgCl_2_, 1 mM EDTA, 1 mM EGTA, and 1 mM DTT, and maintained on ice for 20 minutes. Then, samples were sequentially centrifuged for 5 minutes at 720×g to obtain the nuclear fraction; after that for 20 minutes at 10000×g to obtain the mitochondrial fraction(pellet), and finally for 60 minutes at 100000×g to separate the cytosolic fraction (supernatant) and membrane fraction (pellet). Each fraction was analysed by western-blot as indicated.

### Immunocytochemistry

Neurons were fixed for 20 minutes at RT with 4% paraformaldehyde and washed for 10 minutes in PBS. Fixed cells were incubated with 50 mM NH_4_Cl, blocked and permeabilized for 1 hour with 0.22% gelatin from cold water fish (Sigma) and 0.1% Triton-X-100 in PBS. Cells were incubated with the primary antibodies for 1 h at RT and after 3 washes with blocking solution, the secondary antibodies were added (Invitrogen); nuclei were stained with DAPI 1 µg/ml (Calbiochem). Coverslips were mounted with Fluoromount G (Southern Biotechnology Associates, Inc, Birmingham, Alabama). Photographs were taken with Zeiss Axiovert200 microscope.

### Antibodies and chemicals

Primary antibodies for Akt, Akt pT308, Akt pS473, Akt2, Akt3, GSK3α/β pS21/9, p70 S6K1, pT389, S6, S6 pS235/236, S6 pS240/244, 4EBP1 pT37/46, 4EBP1, 4EBP2, GAPDH, ERK pT202/Y204, p38 MAPK pT180/Y182, p38 MAPK, SAPK/JNK, pT183/Y185, SAPK/JNK, TSC2 pT1462, TSC2, PRAS40 pT246, and PRAS40 were from Cell Signaling (Danvers, Massachussetts); antibodies against Akt1 (PKBα) and PDK1 were from BD Transduction Laboratories, and Lamin B1 and p70 S6K1 were from Santa Cruz Biotech (Santa Cruz, California); primary antibody for Tau-1 was from Chemicon (city, country), against GFP and GSK3α/β were from Invitrogen, and the one for Sin1 was a generous gift of Dr. Alessi's lab [95]. Secondary antibodies for western-blot (horseradish peroxidase conjugated) were from Santa Cruz Biotech and secondary antibodies for immunocytochemistry (Alexa-conjugated) were from Invitrogen.

Akt inhibitor VIII was from Calbiochem and used at 5 µm, and identical amount of DMSO was used as control.

### Viability assay

Cells were treated with 1 µM calcein AM (Invitrogen) and 2 µM propidium iodide for 2 minutes. Photographs were taken with a Leica DM IRE 2 fluorescence microscope.

### Data analysis

All experiments were repeated at least 3 times. Axon and were measured using the NeuronJ 1.4.0 application [96] of ImageJ 1.42j; at least 50 neurons were counted for each condition. The statistical analysis was performed using SigmaPlot 11.0 (Systal Software, Inc).

## Supporting Information

Figure S1 **Evolution of Akt regulators in neuron cultures.** The two main known Akt kinases, PDK1 and mTORC2, were analysed in cortical (left) and hippocampal (right) neuron cultures at DIV 1, 2, 4, 6, 8, and 10. Cell extracts were obtained as described in Methods, and Western blots analysed with specific antibodies against PDK1 and mSin1, a well known mTORC2 component. β-actin was used as load control for cortical neurons and GAPDH for hippocampal neurons. Protein expression was quantified and normalised with respect the control protein as previously indicated. The data from DIV 1 was always considered as 1 relative units and the values are represented in logarithmic scale. The graphs represent three independent experiments (each point represents mean ± SEM). Samples were compared to 1 DIV using Student's t test; *: *p*<0.05; **: *p*<0.01; ***: p<0.001. Note that mSin1 antibody recognized two isoforms, only the large one (80 kDa, marked with an arrow) is part of the mTORC2 complex. The changes observed in Akt phosphorylation did not correlate with changes in these proteins.
(TIF)

Figure S2 **Evolution of Akt substrates implicated in mTORC1 regulation in neuron cultures.** Cortical (**left**) and hippocampal (**right**) neuron cultures were analysed at 1, 2, 4, 6, 8 and 10 DIV. Cell extracts were obtained as described in Methods, and the Western blots analysed with specific antibodies against the indicated proteins. β-actin was used as load control for cortical neurons and GAPDH for hippocampal neurons. Protein expression was quantified and normalised with respect the control protein as indicated. The data from DIV 1 was always considered as 1 relative units and the values are represented in logarithmic scale. The graphs represent three independent experiments (each point represents mean ± SEM). Samples were compared to 1 DIV using Student's t test; *: *p*<0.05; **: *p*<0,01; ***: *p*<0.001. Slight variations were observed for Akt phosphorylated residues of TSC2 and PRAS40 in both cortical and hippocampal neurons. Note that TSC2 suffered a marked decrease in hippocampal neurons along the development.
(TIF)

Figure S3 **Regulation of Akt substrates implicated in mTORC1 by Akt isoforms.** Cortical neurons were infected with lentiviral vectors containing shRNA at two times (1 DIV and 6 DIV) for six hours; proteins were extracted 72 h after infection (at 4 DIV and 9 DIV respectively) and interference was confirmed at the protein level using specific antibodies (see **Figure 6A**). The cell extract was examined using antibodies against the subtrates of Akt implicated in mTORC1 regulation: TSC2, TSC2 pT1462, PRAS40 and PRAS40 pT246. β-actin was used as load control. No statistically significant difference was observed.
(TIF)

Figure S4 **Regulation of MAPKs by Akt isoforms.** Cortical neurons were infected with lentiviral vectors containing shRNA at two times (1 DIV and 6 DIV) for six hours; proteins were extracted 72 h after the infection (at 4 DIV and 9 DIV respectively) and interference was confirmed at the protein level using specific antibodies (see **Figure 6A**). The cell extract was examined using antibodies against the reporters of the main MAPK pathways activation: ERK1/2, ERK1/2 pT202/Y204, JNK, JNK pT183/Y185, p38 and p38 pT180/Y182. No statistically significant difference was observed.
(TIF)
