# Supplementary figures and images for: Specific Roles of Akt iso Forms in Apoptosis and Axon Growth Regulation in Neurons
Source: PLoS One. 2012 Apr 11;7(4):e32715. doi: 10.1371/journal.pone.0032715 (PMC3324480; doi:10.1371/journal.pone.0032715)

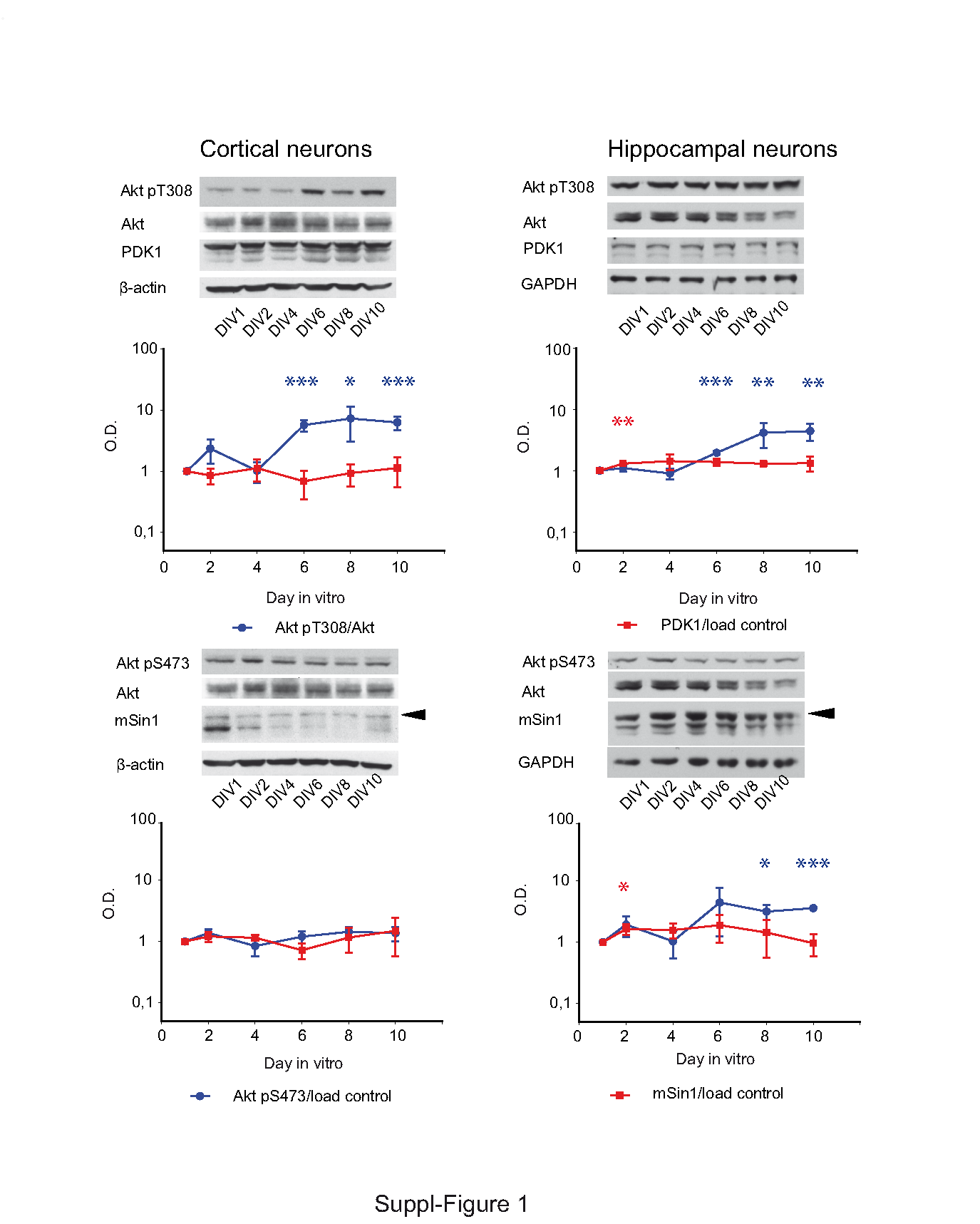

Supplement: Figure S1 — Evolution of Akt regulators in neuron cultures. The two main known Akt kinases, PDK1 and mTORC2, were analysed in cortical (left) and hippocampal (right) neuron cultures at DIV 1, 2, 4, 6, 8, and 10. Cell extracts were obtained as described in Methods, and Western blots analysed with specific antibodies against PDK1 and mSin1, a well known mTORC2 component. β-actin was used as load control for cortical neurons and GAPDH for hippocampal neurons. Protein expression was quantified and normalised with respect the control protein as previously indicated. The data from DIV 1 was always considered as 1 relative units and the values are represented in logarithmic scale. The graphs represent three independent experiments (each point represents mean ± SEM). Samples were compared to 1 DIV using Student's t test; *: p<0.05; **: p<0.01; ***: p<0.001. Note that mSin1 antibody recognized two isoforms, only the large one (80 kDa, marked with an arrow) is part of the mTORC2 complex. The changes observed in Akt phosphorylation did not correlate with changes in these proteins. (TIF) [file pone.0032715.s001.tif]

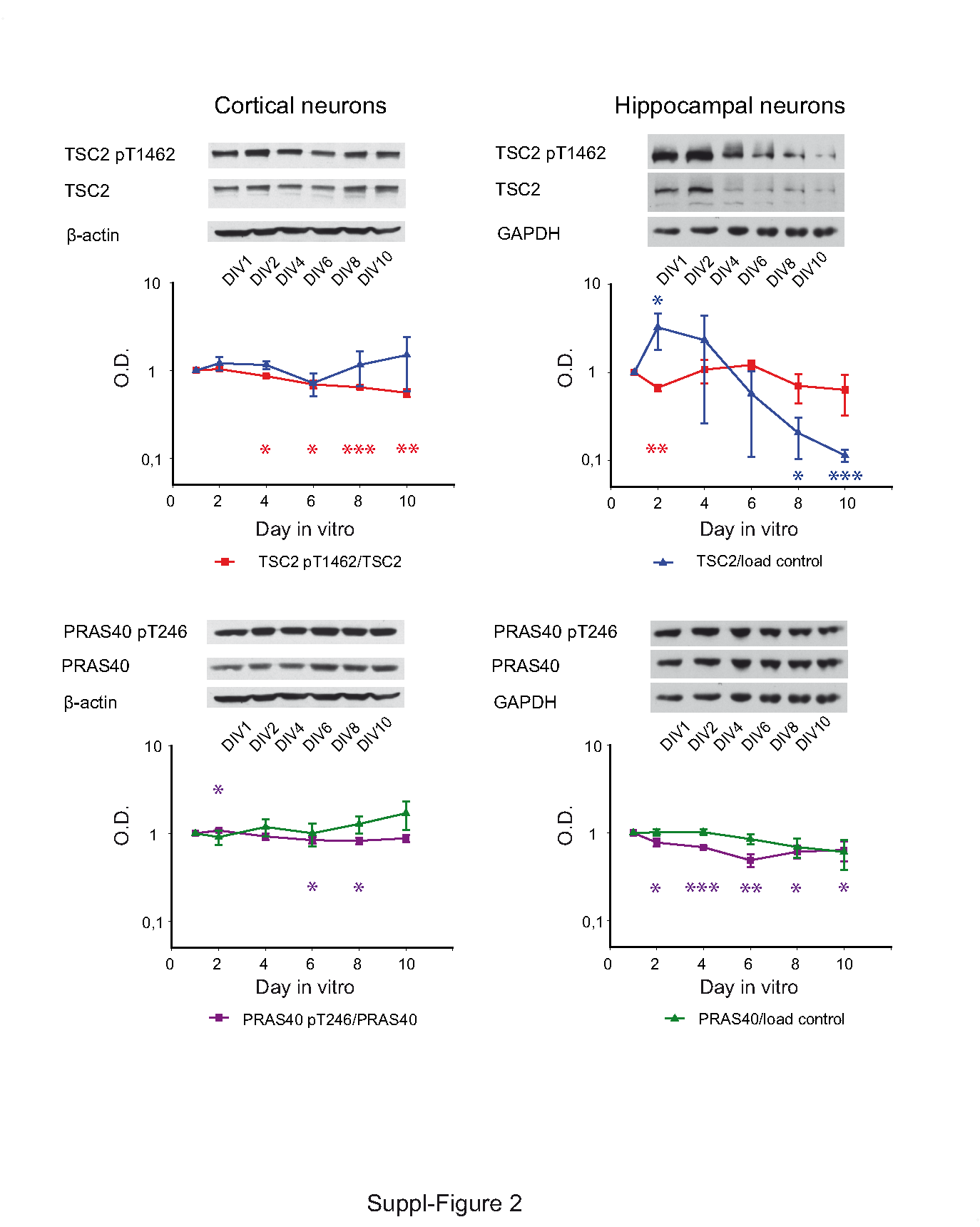

Supplement: Figure S2 — Evolution of Akt substrates implicated in mTORC1 regulation in neuron cultures. Cortical (left) and hippocampal (right) neuron cultures were analysed at 1, 2, 4, 6, 8 and 10 DIV. Cell extracts were obtained as described in Methods, and the Western blots analysed with specific antibodies against the indicated proteins. β-actin was used as load control for cortical neurons and GAPDH for hippocampal neurons. Protein expression was quantified and normalised with respect the control protein as indicated. The data from DIV 1 was always considered as 1 relative units and the values are represented in logarithmic scale. The graphs represent three independent experiments (each point represents mean ± SEM). Samples were compared to 1 DIV using Student's t test; *: p<0.05; **: p<0,01; ***: p<0.001. Slight variations were observed for Akt phosphorylated residues of TSC2 and PRAS40 in both cortical and hippocampal neurons. Note that TSC2 suffered a marked decrease in hippocampal neurons along the development. (TIF) [file pone.0032715.s002.tif]

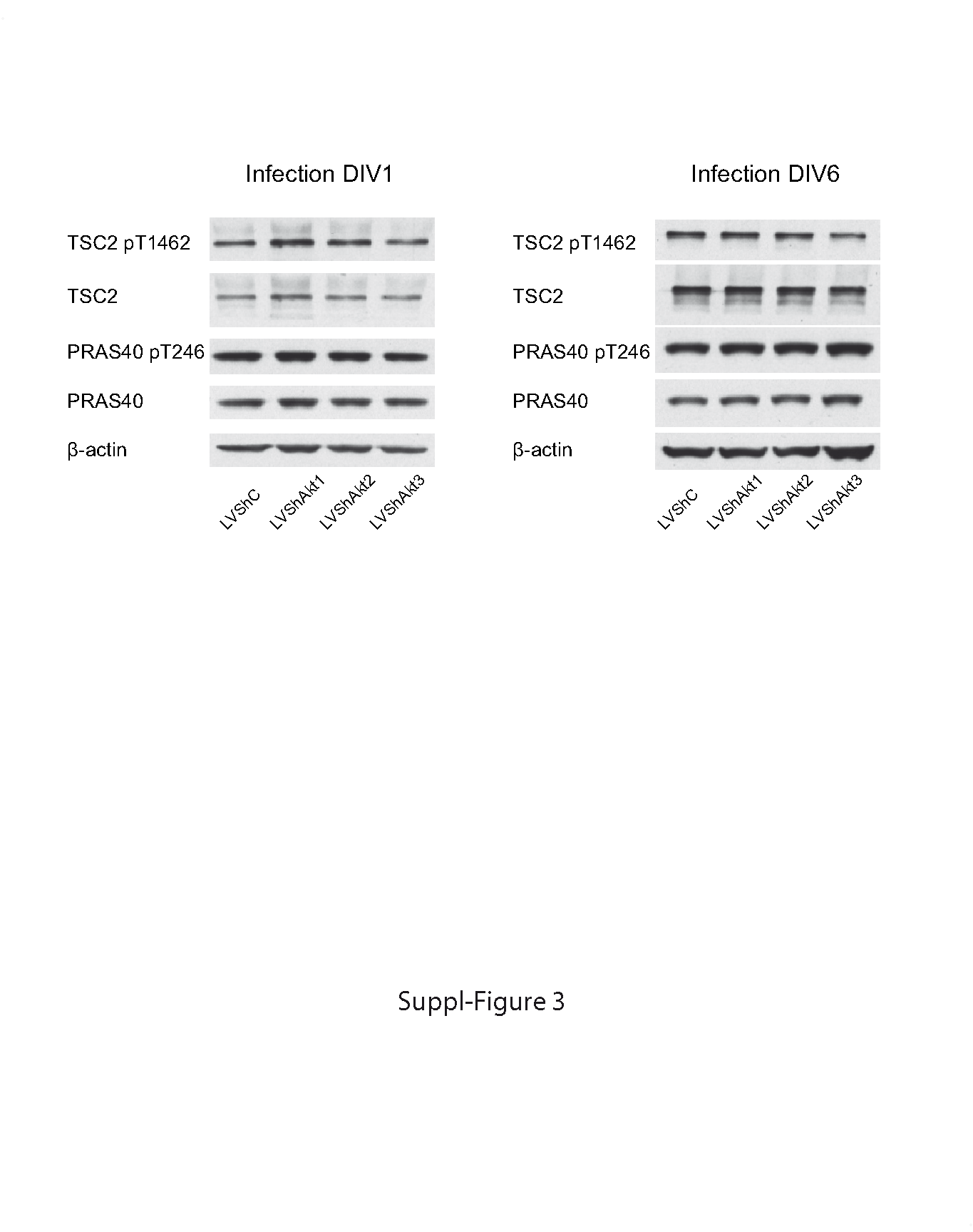

Supplement: Figure S3 — Regulation of Akt substrates implicated in mTORC1 by Akt isoforms. Cortical neurons were infected with lentiviral vectors containing shRNA at two times (1 DIV and 6 DIV) for six hours; proteins were extracted 72 h after infection (at 4 DIV and 9 DIV respectively) and interference was confirmed at the protein level using specific antibodies (see Figure 6A ). The cell extract was examined using antibodies against the subtrates of Akt implicated in mTORC1 regulation: TSC2, TSC2 pT1462, PRAS40 and PRAS40 pT246. β-actin was used as load control. No statistically significant difference was observed. (TIF) [file pone.0032715.s003.tif]

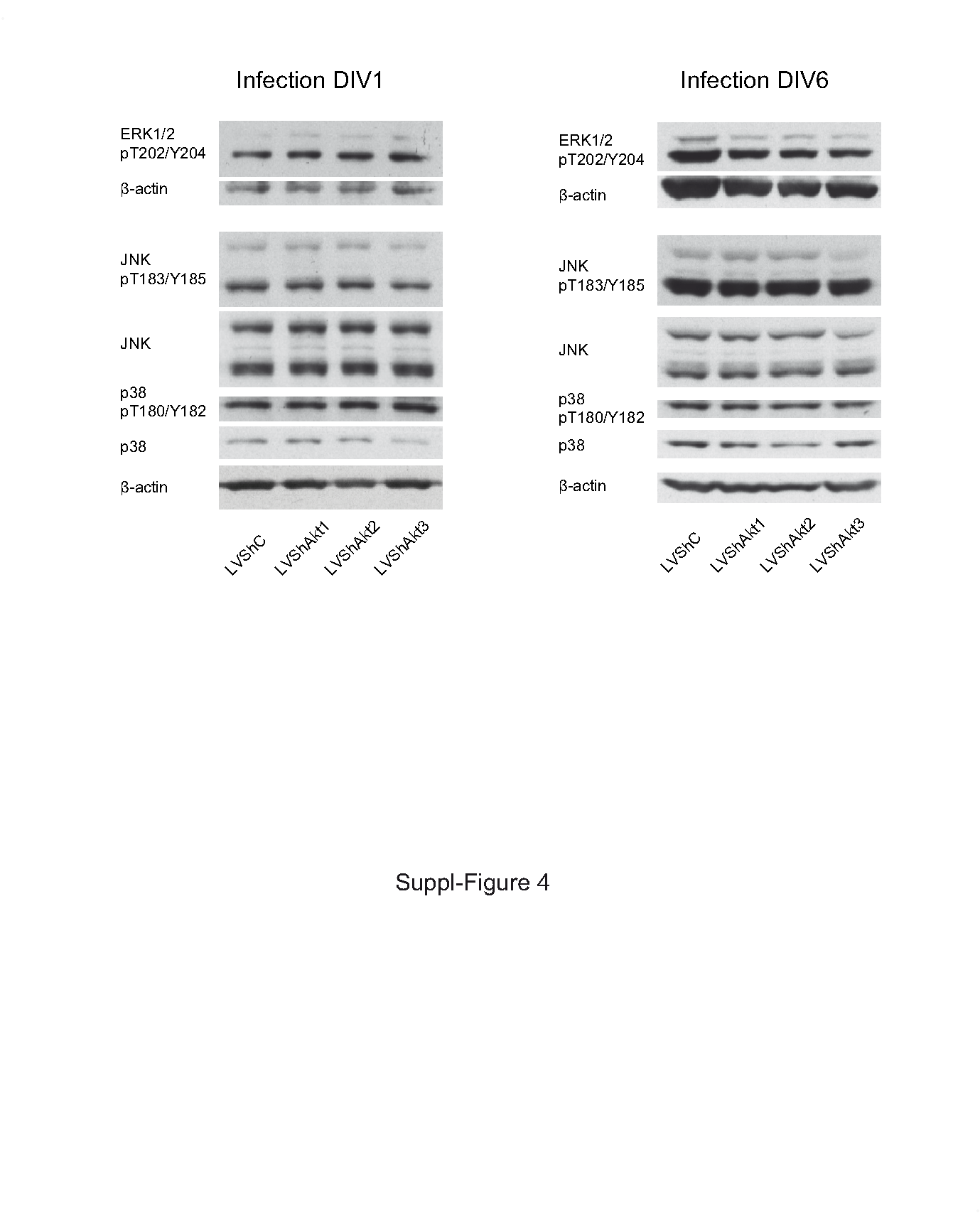

Supplement: Figure S4 — Regulation of MAPKs by Akt isoforms. Cortical neurons were infected with lentiviral vectors containing shRNA at two times (1 DIV and 6 DIV) for six hours; proteins were extracted 72 h after the infection (at 4 DIV and 9 DIV respectively) and interference was confirmed at the protein level using specific antibodies (see Figure 6A ). The cell extract was examined using antibodies against the reporters of the main MAPK pathways activation: ERK1/2, ERK1/2 pT202/Y204, JNK, JNK pT183/Y185, p38 and p38 pT180/Y182. No statistically significant difference was observed. (TIF) [file pone.0032715.s004.tif]
